# The trajectory of putative astroglial dysfunction in first episode schizophrenia: a longitudinal 7-Tesla MRS study

**DOI:** 10.1038/s41598-021-01773-7

**Published:** 2021-11-16

**Authors:** Peter Jeon, Michael Mackinley, Jean Théberge, Lena Palaniyappan

**Affiliations:** 1 Department of Medical Biophysics, Western University, London, Canada; 2 Imaging Division, Lawson Health Research Institute, London, Canada; 3 Robarts Research Institute, Western University, London, Canada; 4 Department of Neuroscience, Western University, London, Canada; 5 Diagnostic Imaging, St. Joseph’s Health Care, London, Canada; 6 Department of Medical Imaging, Western University, London, Canada; 7 Department of Psychiatry, Western University, London, Canada; 8 Robarts Research Institute, UWO, 1151 Richmond Street N., Room 3208, London, ON N6A 5B7 Canada

**Keywords:** Neurochemistry, Predictive markers, Schizophrenia

## Abstract

Myo-inositol is mainly found in astroglia and its levels has been shown to be reduced in the anterior cingulate cortex (ACC) of patients with schizophrenia. We investigate the status of astroglial integrity indexed by ACC myo-inositol at the onset and over the first 6 months of treatment of first episode schizophrenia. We employed 7 T magnetic resonance spectroscopy (1H-MRS) and quantified myo-inositol spectra at the dorsal ACC in 31 participants; 21 patients with schizophrenia with median lifetime antipsychotic exposure of less than 3 days, followed up after 6 months of treatment, and 10 healthy subjects scanned twice over the same period. We studied the time by group interaction for myo-inositol after adjusting for gender and age. We report significant reduction in myo-inositol concentration in the ACC in schizophrenia at an early, untreated state of acute illness that becomes insignificant over time, after instituting early intervention. This trajectory indicates that dynamic astroglial changes are likely to operate in the early stages of schizophrenia. MRS myo-inositol may be a critical marker of amelioration of active psychosis in early stages of schizophrenia.

## Introduction

Astrocytes appear to have many roles that are critical for the pathophysiology of schizophrenia^1–3^. In addition to their contribution to the redox balance in the brain^4,5^, they also clear extracellular glutamate from synaptic space^6,7^, maintain synaptic integrity and support myelination^8–10^, all of which are critical pieces in the mechanistic pathways suspected in schizophrenia^10–12^.

In vivo imaging of astrocytic function has been challenging as direct measures of astroglial activity are elusive. While not a direct marker of cellular activity, magnetic resonance spectroscopy (1H-MRS) allows the measurement of myo-inositol, a compound that is particularly abundant in astroglia than any other brain cells^13,14^. When astrocyte cells are activated due to excessive demands, increase in MRS myo-inositol resonance occurs^15,16^. An increase in myo-inositol also occurs as a response to brain injury^17,18^, in the form of astrogliosis^19,20^. The presence of lower than normal levels of myo-inositol may indicate an insufficient astroglial activity, and thus a state of vulnerability for redox imbalance, excitotoxicity, as well as a reduced ability to mount an appropriate astroglial-mediated inflammatory defence in the face of adversity^1^. A longitudinal reduction in myo-inositol levels over the course of an illness may indicate progressive astroglial pathology (see Supplementary Materials for more discussion on myo-inositol as a proxy for astroglial integrity).

In our previous work, we synthesized MRS studies reporting myo-inositol resonance in schizophrenia and reported a significant but small effect-size reduction of myo-inositol in the anterior cingulate cortex (ACC) in patients with schizophrenia^21^. To date, most of the studies have investigated patients with long-term exposure to antipsychotics. The only exception is Théberge et al.^22^, who did not observe any baseline differences in myo-inositol in unmedicated patients when examining small 1.5 cc voxels using a 4 T MRS approach reported to have a 35% smallest detectable difference for myo-inositol. The 10-months follow-up data in their study was available only for 13 patients, thus limiting the ability to detect longitudinal changes.

In this study, we tested if (1) astroglial dysfunction deficit indexed by ACC MRS measure of myo-inositol is present in early stages of psychosis and (2) whether this deficit progressively worsens or improves in the first 6 months of treatment. In line with our previous work, we expect to observe a significant reduction in MRS myo-inositol levels in patients with first episode schizophrenia compared to a cohort of healthy controls and that this deficit would improve significantly over the first 6 months of treatment. To our knowledge, this is the first longitudinal report of myo-inositol from 7 T MRS in schizophrenia.

## Results

### Demographic data

Demographic and clinical data of subjects are shown in Table 1. As reported previously, the DUP (mean) was 29 weeks (SD = 26 weeks) and the duration of antipsychotic use was < 3 days prior to the first scan session. SOFAS scores were significantly different between the 2 groups (t(29) = 12.466, *P* < 0.001). The time in between baseline and follow-up (FUP) scan was 5.9 months (SD = 1.3 months) for patients and 7.3 months (SD = 1.9 months) for healthy controls (HC).

**Table 1:** Demographic and clinical characteristics.

Characteristic	Patient group (*N* = 21)	Healthy controls (*N* = 10)	*t/χ*^2^	*P*
Gender (male/female)	16/5	5/5	2.13	0.145
Marital status (Mar/S)	1/20	1/9	0.31	0.58
Inpatient at baseline (Y/N)	11/10			
Family Hx (Y/N/DN)	10/6/5			
AP exposure at baseline (M/SD; days)	2.95/3.11			
Total DDD-days at baseline scan (M/SD)	2.25/4.74			
Total DDD-days at FUP scan (M/SD)	145.68/97.56			
DUP (M/SD/median; weeks)	29.38/26.65/18			
Ethnicity (Black/White/Other)	2/18/1	0/5/5	4.51	0.034^a^
Age (M/SD)	22.33/5.29	21.60/3.37	− 0.47	0.645
SOFAS at baseline scan (M/SD)	42.33/12.84	83.70/5.62	12.47	0.000
SOFAS at FUP scan (M/SD)	61.25/9.85	85.10/3.21	9.83	0.000
PANSS-8 total at baseline scan (M/SD)	24.67/5.30			
PANSS-8 total at FUP scan (M/SD)	14.35/4.77			
Smoker (yes/no)	6/15	0/10	3.54	0.060
Cannabis user (yes/no)	13/8	0/10	10.66	0.001
Time between scans (M/SD; months)	5.93/1.25	7.67/1.90	2.63	0.021

*Mar* married, *S* single, *Y* yes, *N* no, *Hx* history, *DN* don’t know, *AP* antipsychotic, *M* mean, *SD* standard deviation, *DDD* defined daily dose, *FUP* follow-up, *DUP* duration untreated psychosis.

*P* values for differences between groups were calculated using chi-square analyses for categorical variables and independent *t* tests for continuous variables.

^a^White vs non-White comparison.

**Figure 1:**
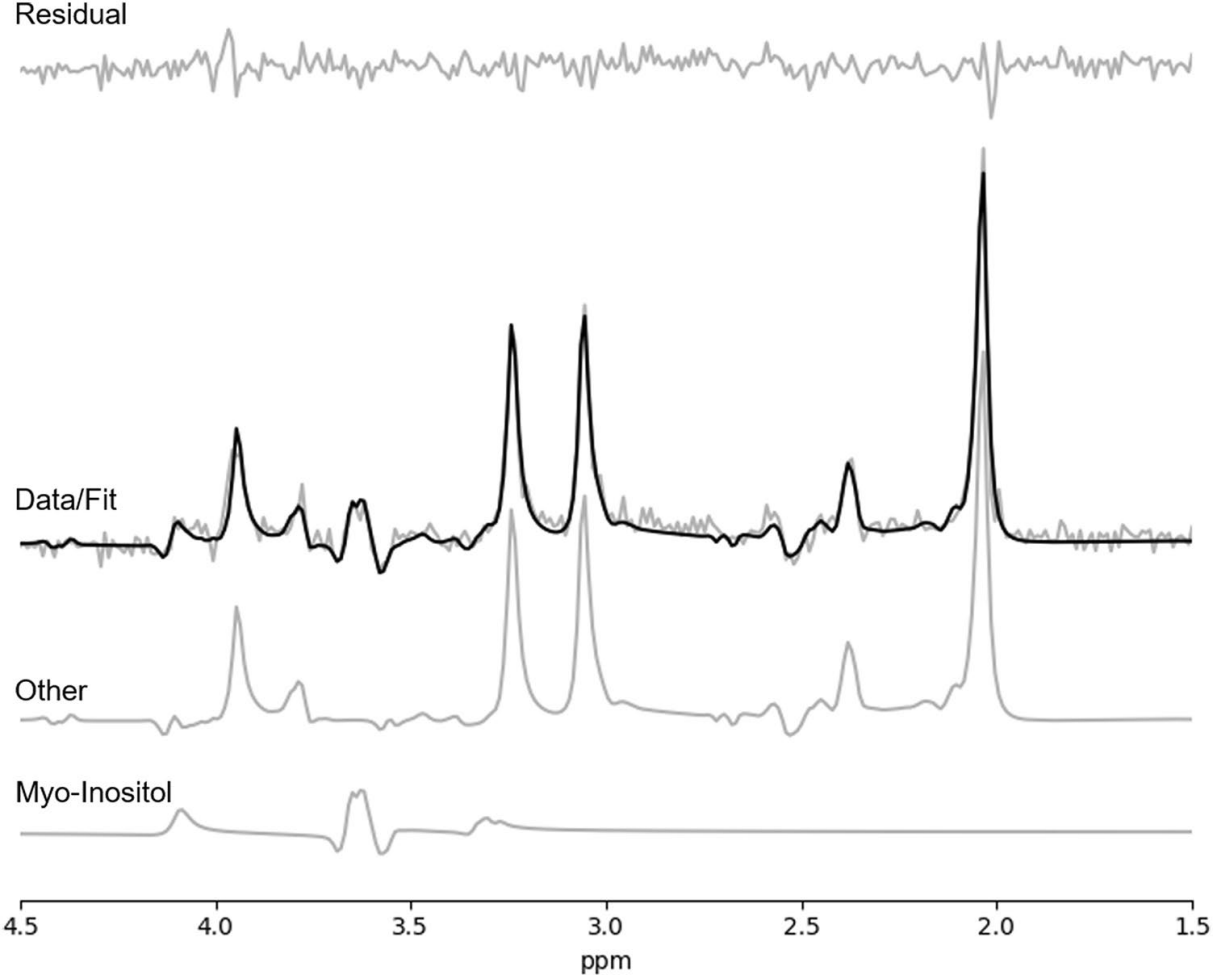
Sample fitted spectrum of a single participant. Fit spectrum (bolded) is overlaid on the raw spectrum with the residual spectrum displayed above. The myo-inositol spectra and the sum of the remaining 16 template-included metabolites (labeled as ‘other’) are displayed below.

Cramer-Rao lower bounds (CRLB) values indicating the quality of myo-inositol measurement was quantified for both groups. Myo-inositol CRLB values for HC and FES were 4% (SD = 1%) for both baseline and FUP. Thus the 2 groups had acceptable qualitative metrics for myo-inositol estimation at both time points. A sample of fitted spectrum is presented in Fig. 1. We present the concentrations and CRLBs of metabolites other than myo-inositol in the Supplementary Materials.

### Myo-inositol levels

**Figure 2:**
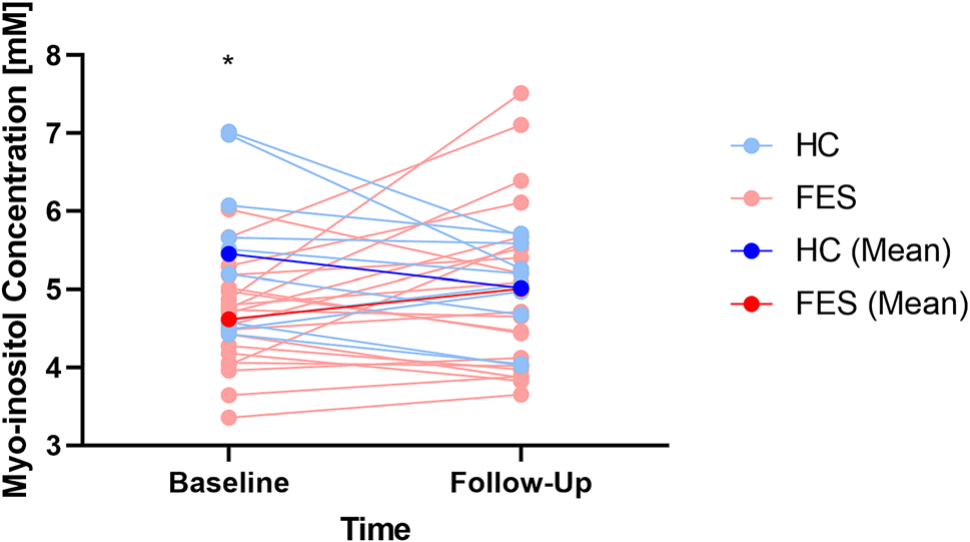
Means of myo-inositol [mM] for healthy controls (HC, blue) and patients (FES, red) at baseline and follow-up scan sessions. Subject-level myo-inositol changes are shown in light blue for healthy controls and pink for patients. Asterisk denotes significant difference between means (Note: y-axis values do not begin at 0 for improving data visualization).

Repeated measures ANOVA revealed a significant group × time interaction (F(1,27) = 6.40, *P* = 0.018, partial eta-squared pη2 = 0.19) as well as a group effect (F(1,27) = 8.08, *P* = 0.008, pη2 = 0.23), between FES (M = 4.72 mM, SD = 0.64 mM) and HC (M = 5.44 mM, SD = 0.65 mM) but no effect on time (F(1,27) = 0.05, *P* = 0.83). Parameter estimates revealed that at baseline, FES had lower myo-inositol than healthy controls (t(29) = 4.88, *P* < 0.001), but this difference was not present at follow-up (t(29) = 0.78, *P* = 0.44). A simple contrast of time in each group revealed no significant effect in both the healthy control group (F(1,7) = 0.34, *P* = 0.58; 8.2% decrease) and in patients (F(1,18) = 0.28, *P* = 0.60; 8.9% increase) (Fig. 2). Younger age and female gender were associated with lower levels at both time points (F(1,27) = 8.1–11.2, pη2 = 0.14–0.36, *P* = 0.06–0.001).

Lastly, the time interval between scans in months was not related to myo-inositol concentration differences (FUP-baseline) in either group (HC: *r* = 0.005, *P* = 0.90; FES: *r* =  − 0.096, *P* = 0.68). Nevertheless, to address spurious differences in change scores that may arise due to time differences in between scans, we generated time-adjusted, i.e. annualized myo-inositol concentration values. Annualized change scores in myo-inositol change were significantly different between the two groups (t(29) =  − 2.50, *P* = 0.018) and indicated a 0.19 mM/year (SD = 0.37 mM) increase in patients and a 0.12 mM/year (SD = 0.15 mM) reduction in healthy controls.

### Myo-inositol and clinical measures

**Figure 3:**
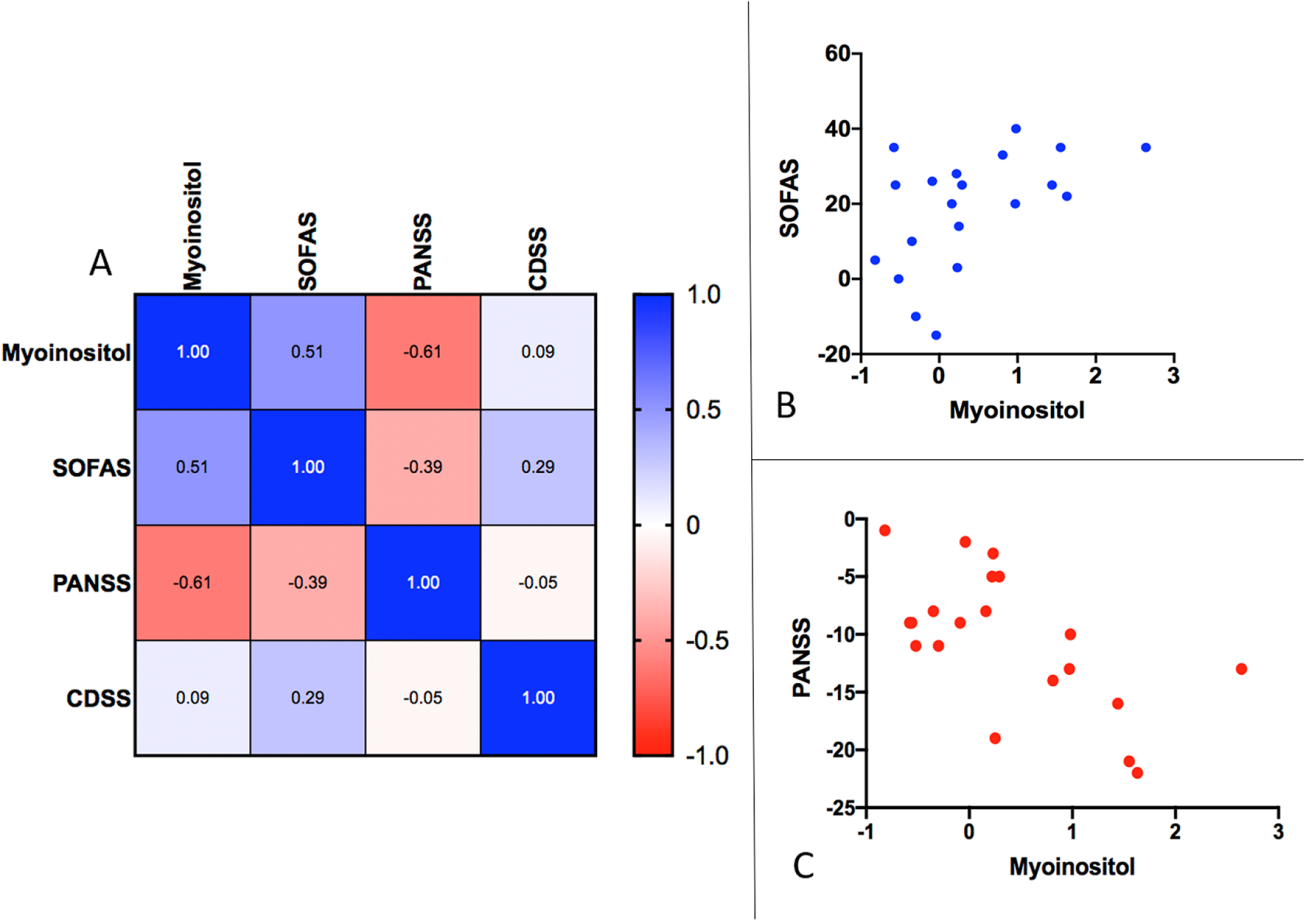
Relationship between longitudinal change in myo-inositol levels and illness burden. (**A**) Heat map of linear correlations among change values (baseline to 6-months follow-up). (**B**) Correlation plot of the relationship between SOFAS and myo-inositol change scores. (**C**) Correlation plot of the relationship between PANSS-8 and myo-inositol change scores. Images were generated using GraphPad Prism version 9 for Windows (GraphPad Software, San Diego, California USA, www.graphpad.com). *CDSS* Calgary depression scale in schizophrenia, *PANSS-8* positive and negative symptom scale—8 item version, *SOFAS* Social and Occupational Functioning Scale.

As myo-inositol concentration increased in patients, PANSS-8 scores reduced significantly (Spearman’s *rho* =  − 0.61, *P* = 0.004) and SOFAS increased marginally (*rho* = 0.44, *P* = 0.05), but changes in myo-inositol did not track changes in depressive burden measured using CDSS (*rho* = 0.001, *P* = 0.90) (Fig. 3). There was no significant correlation between annualized myo-inositol concentration changes and DUP (*rho* = 0.05, *P* = 0.83). We also did not see any correlation between baseline (unadjusted) myo-inositol concentration and DUP (*rho* = 0.30, *P* = 0.22), SOFAS (*rho* = − 0.27, *P* = 0.23), CDSS (*rho* = − 0.10, *P* = 0.65), or PANSS-8 total (*rho* = 0.18, *P* = 0.43).

There was no significant relationship between baseline antipsychotic exposure defined daily dose (DDD) myo-inositol levels (*rho* = − 0.15, *P* = 0.51) and DDD at follow-up and myo-inositol (*rho* =  − 0.06, *P* = 0.81) as well as between DDD at follow-up and annualized myo-inositol change (*rho* =  − 0.14, *P* = 0.56). See the Supplementary Materials for the association between the change scores in myo-inositol and glutamate, a putative marker of the metabolic integrity of excitatory neurons.

To examine the effect of injectables vs. oral atypical antipsychotics on follow-up levels of myo-inositol, we studied the effects of using long-acting injectables (n = 8) vs. oral antipsychotics (n = 11). There was no significant difference in myo-inositol levels at 6-months (LAI users mean (SD) = 4.67(0.96); non-users mean (SD) = 5.35(1.20); t(17) = − 1.38, *P* = 0.19). To examine the effect of partial agonists vs. D2-blockers on clinical outcome, we studied the effects of using partial agonists (aripiprazole and brexpiprazole, n = 10) vs. other D2-antagonists (n = 9) on the myo-inositol levels at 6-months. There was no significant difference in myo-inositol levels at 6-months (partial agonists mean (SD) = 4.98(0.89); D2-antagonists mean (SD) = 5.16(1.41); t(17) = -0.34, *P* = 0.74). A detailed list of patient antipsychotic drug information can be found in the Supplementary Materials.

## Discussion

We report 3 major observations in this study: (1) astroglial dysfunction indexed by reduced ACC MRS myo-inositol resonance is present in early stages of psychosis and (2) this effect of diagnosis is limited to the untreated stage of illness and (3) improvement in myo-inositol levels relate to decreasing burden of psychotic symptom severity and improving social and occupational functioning. Taken together, this longitudinal report of myo-inositol resonance from 7 T MRS in first episode schizophrenia supports a role for early astroglial dysfunction that may track symptom burden and functional outcomes of the illness. As reported in the Supplementary Materials, the longitudinal changes in myo-inositol are also statistically associated with the changes in glutamate levels. This indicates that a shared process may underlie the changes in neuro-metabolites that are subject to astroglial regulation in early stages of schizophrenia.

In contrast to Chiappelli et al.^23^, we did not observe a relationship between myo-inositol resonance and depressive burden in our sample. Our sample excluded patients with affective psychosis and exclusively consisted of untreated individuals, unlike the sample recruited by Chiappelli and colleagues. In contrast, we noted a significant correlation between symptom and function improvement and increasing myo-inositol values. Plitman et al.^24^ did not observe a similar relationship between symptom burden and myo-inositol levels, though their measurement was restricted to associative striatum. It is likely that the critical changes in astroglia are regionally distinct, and likely involve ACC preferentially^25–27^.

We report a significant group effect indicating myo-inositol reduction in FES; this is consistent with the meta-analysis of numerous MRS studies reporting a reduction of small effect-size in established schizophrenia^21^. The lack of group differences in myo-inositol levels that we report in patients at follow-up may indicate a process of normalisation with clinical improvement in early stages. Notable fluctuations can occur in in myo-inositol levels over time, even in the absence of a disease process, as demonstrated by an equivalent magnitude of the effect of time in the healthy control group. Given that the main source of myo-inositol in human brain is the phosphoinositide pathway involving the cell membranes^28^, many physiological conditions that affect the membrane properties can affect myo-inositol concentration (e.g., plasma sodium levels^29^, osmolality^30^, diet patterns^31^, sleep deprivation^32^, exercise habits^33^). Changes in the biophysical properties of the cell membrane induces rapid and reversible changes in brain myo-inositol at a much shorter time scale than the one employed in our study^34^. In this context, the clinical correlations reported here are best considered as one of many factors that influence these fluctuations in the patient group. On the other hand, in disorders such as Alzheimer’s disease, a longitudinal increase in myo-inositol occurs in relation to increased severity of neurodegenerative changes^35,36^. Further longitudinal studies with multiple time points of measurements over longer periods of time are warranted to clarify this issue in psychosis.

Some of the notable strengths of our study include the use of 7 T scanner, and recruiting highly symptomatic, mostly drug-naïve individuals. Some of the limitations include the use of a single voxel (dorsal ACC) and a single time point (6 months) for follow-up; as a result, we cannot exclude the possibility of increased or preserved levels of myo-inositol in other brain regions or at ACC at the later stages of the illness. It is worth noting that myo-inositol participates in several neuronal and astroglial cellular processes, involving glucose and lipid metabolism and ion channel functions^37^. Intracellular myo-inositol levels can be altered by a wide variety of metabolic impairments^37^ as well as the use of mood stabilisers^38^. Our interpretation of myo-inositol resonance as a reflection of astroglial integrity is in line with the majority of MRS literature to date^39–41^; nevertheless, it should be only considered as a proxy marker of astroglial integrity. Furthermore, astrocyte count and thus myo-inositol resonance, may reduce with antipsychotic exposure, as shown in macaques exposed to haloperidol or olanzapine^42^. This effect is controversial, as antipsychotic-induced increase in astroglial density has been reported in rats^43^ and rhesus monkeys^44^. Drug-induced astrocyte depletion, if present in our sample, would have further reduced the myo-inositol levels at 6-months follow-up; but we observed lower values in patients than controls at the baseline, before exposure to antipsychotics was established. This is further supported by the lack of treatment-induced changes on MRS myo-inositol levels reported in a recent meta-analysis across various stages of schizophrenia^45^. Finally, though our sample size, especially for healthy controls was limited, this was sufficient to detect the postulated Group × Time interaction.

In summary, this longitudinal 7 T MRS study of ACC confirms the presence of reduced myo-inositol in the early stages of schizophrenia, that may progressively improve in patients who also show symptomatic improvement. This supports the possibility of a putative astroglial deficit predating the first presentation of psychosis, likely of early developmental^46^ or of inflammatory^47^ origin, but showing a trend of reversal with early intervention. Further, this also raises the need for experimental studies targeting the putative astroglial dysfunction, as this may improve clinical and functional outcomes in psychosis.

## Methods

This sample has been previously reported^48^, and comprises 21 volunteers with first-episode schizophrenia (FES) and 10 healthy volunteers. Healthy controls were recruited by word-of-mouth and group-matched for age, gender, and parental socio-economic status. Both groups were studied around the same time (Supplementary Table S1). Patients were required to have < 14 days of lifetime exposure to antipsychotics and satisfy a consensus diagnosis of first-episode schizophrenia by 3 psychiatrists after 6 months of program entry, based on the DSM-5 criteria^49^ and Leckman’s best estimate procedure^50^. Any participant whose 6-month diagnoses were bipolar or major depressive disorder with psychoses, as well as suspected drug-induced psychoses, were excluded from the study. All patients were recruited from the PEPP (Prevention and Early Intervention for Psychosis Program) at London Health Sciences Centre and received routine care, in line with the Canadian standards for early intervention in psychosis^51^. Healthy volunteers were screened to ensure to have no personal history of mental illness and no family history of psychotic disorder. None of the participants had a significant head injury, major medical illness, or MRI contraindications. All subjects provided written, informed consent according to the guidelines of the Human Research Ethics Board for Health Sciences at Western University, London, Ontario. The study was conducted according to the guidelines of the Declaration of Helsinki and approved by the Western University Research Ethics Board of the University of Western Ontario.

### MRS acquisition and analysis

MRS measurements were acquired using a Siemens MAGNETOM 7 T head-only MRI scanner (Siemens, Erlangen, Germany) and a site-built head coil (8-channel transmit, 32-channel receive) at the Centre for Functional and Metabolic Mapping of Western University (London, Ontario). This acquisition has been employed in our prior MRS studies^48,52,53^. Further details of the acquisition and spectral fitting are described in the Supplementary Materials.

### Clinical assessment

The following clinical assessments were undertaken on the same day of scanning:

1. Psychotic symptom severity measured using PANSS-8^54,55^ scale.
2. The overall social and occupational functioning at the time of first presentation using SOFAS^56^.
3. Depressive burden measured using Calgary Depression Scale in Schizophrenia (CDSS).

In addition, we also assessed the duration of untreated psychosis (DUP) using multiple sources of information provided by the patient, the referring sources, and caregivers as well as by reviewing clinical charts. We used the first emergence of positive psychotic symptoms as the starting point for calculating the DUP, in line with prior work^57^.

### Statistical analyses

All statistical tests were computed using IBM SPSS Statistics version 26^58^. Group demographic differences were calculated using *t* tests and chi-square tests for continuous and dichotomous variables, respectively. Repeated measures ANOVA was used to assess group × time interaction (primary hypothesis), as well as group effect and time effect, with parameter estimates examined to test individual group effects. As age and gender are known modifiers of myo-inositol levels, they were entered as covariates in the ANOVA model. When correlating changes in myo-inositol to clinical features, all changes scores were calculated as (follow-up—baseline), in the same direction for all variables.

## Supplementary Information

Supplementary Information.
