# Supplementary material for: The trajectory of putative astroglial dysfunction in first episode schizophrenia: a longitudinal 7-Tesla MRS study
Source: Sci Rep. 2021 Nov 16;11:22333. doi: 10.1038/s41598-021-01773-7 (PMC8595701; doi:10.1038/s41598-021-01773-7)
Supplement: Supplementary file 1 — Supplementary Information. [file 41598_2021_1773_MOESM1_ESM.pdf]

Supplementary Materials

Myo-inositol as a Proxy for Astroglial Integrity 1
Participant Scan Dates 2
MRS Acquisition and Spectral Fitting 2
Spectral Fit Quality and Metabolite Quantification 4
Association Between Longitudinal Myo-inositol and Glutamate 4
Myo-inositol and Cannabis 4
Patient Antipsychotic Drug Information 4
Supplementary Table 1 5
Supplementary Figure 1 6
Supplementary Table 2 7
Supplementary Figure 2 8
Supplementary Figure 3 9
Supplementary Figure 4 9
Supplementary Table 3 10
References 13

Myo-inositol as a Proxy for Astroglial Integrity

Studies linking myo-inositol to astroglial activation in humans are sparse. Rothermundt and colleagues (2007)[1] demonstrated concurrent increase in serum S100B concentrations and elevated myo-inositol in the brain. When demyelinating lesions in patients with Multiple Sclerosis obtained via stereotactic needle brain biopsies stain positive for GFAP, indicating astrogliosis, concomitant elevation of myo-inositol resonance (84-160%) occurs in the lesion site [2]. Another line of evidence comes from the study of malignant gliomas of the human brain. High levels of myo-inositol are often seen in low-grade gliomas with malignant proliferation of astrocytes, before cellular destruction occurs (due to infarction or radiation damage from treatment) [3,4].

Animal studies have been more relevant to address this issue. Filibian and colleagues (2012) measured myo-inositol from hippocampus of alive anaesthetised rats using ^{1}H-MRS and validated this measure using post-mortem cell-specific markers stained for glial fibrillary acidic protein (GFAP) and S100B. Chen and colleagues (2009)[5] studied transgenic mice in comparison with wild types and reported increase in MRS myo-inositol in conjunction with histopathological findings of activation and proliferation of astrocytes in frontal cortex and hippocampus. More direct evidence was later provided by the same group [6], by experimentally inducing neural proliferation and astrocyte loss using a neural stem-cell transplant; a reduction in the number of astrocytes was reflected in a reduction in MRS myo-inositol resonance, 6 weeks after the transplant.

Participant Scan Dates

Supplementary Table 1 indicates the baseline and follow-up scan dates for all participants. The scan dates for the patient group ranged from March 28, 2017 to November 23, 2018 while the scan dates for the control group ranged from February 10, 2017 to September 10, 2018.

MRS Acquisition and Spectral Fitting

A two-dimensional sagittal anatomical image (37 slices, TR = 8000 ms, TE = 70 ms, flip-angle (α) = 120°, thickness = 3.5 mm, field of view = 240×191 mm) was used as reference to prescribe a 2.0 x 2.0 x 2.0 cm (8 cm^3) ^{1}H-MRS voxel on the bilateral dorsal ACC (Supplementary Figure 1). Voxel positioning was set by having the posterior end of the voxel coinciding with the precentral gyrus and the caudal face of the voxel coinciding with the most caudal positioning that was not part of the corpus callosum. Voxel angle was set to be tangential to the corpus callosum. A semi-LASER ^{1}H-MRS sequence (TR = 7500 ms, TE = 100 ms, bandwidth = 6000 Hz, N = 2048) was used to acquire 32 channel-combined, VAPOR [7] water-suppressed spectra as well as a water-

unsuppressed spectrum to be used for spectral post-processing, fitting and quantification. During scan, participants were asked to rest by fixing their gaze on a white cross on a 50% gray background.

Using the tools outlined in Near et al. [8], the 32 spectra were phase and frequency corrected before being averaged into a single spectrum to be used for all subsequent analyses. QUECC [9] and HSVD [10] were applied to the spectrum for lineshape deconvolution and removal of residual water signal, respectively. Spectral fitting was done using fitMAN [11], a time-domain fitting algorithm that uses a non-linear, iterative Levenberg-Marquardt minimization algorithm to estimate the chemical shift, amplitude, linewidth and phase (0th and 1st order) of echo time-specific prior knowledge templates. The metabolite fitting template included 17 brain metabolites: alanine, aspartate, choline, creatine, γ-aminobutyric acid (GABA), glucose, glutamate, glutamine, glutathione, glycine, lactate, myo-inositol, N-acetyl aspartate, N-acetyl aspartyl glutamate, phosphorylethanolamine, scyllo-inositol, and taurine. No significant macromolecule contribution was expected due to the long echo time and hence was omitted from the metabolite template. Metabolite quantification was then performed using Barstool [12] with corrections made for tissue-specific (gray matter, white matter, CSF) T_1 and T_2 relaxations through partial volume segmentation calculations of voxels mapped onto T_1-weighted images acquired using a 0.75 mm isotropic MP2RAGE sequence (TR = 6000 ms, TI_1 = 800 ms, TI_2 = 2700 ms, flip-angle 1 (α_1) = 4°, flip-angle 2 (α_2) = 5°, FOV = 350 mm × 263 mm × 350 mm, T_{acq} = 9 min 38 s, $iPAT_{PE}$ = 3 and 6/8 partial k-space). All spectral fit underwent visual quality inspection as well as Cramer-Rao lower bounds (CRLB) assessment for each metabolite.

Spectral Fit Quality and Metabolite Quantification

Spectral fit quality for each metabolite in our template was assessed by Cramer-Rao lower bound (CRLB) percentage values (Supplementary Table 2). Out of 17 metabolites included in the fitting template, eight metabolites are reported here that met the individual CRLB cut-off of 50%. A sample fitted spectrum outlining all metabolites included in our template is presented in Supplementary Figure 2.

Association Between Longitudinal Myo-inositol and Glutamate

A positive correlation was found between annualized myo-inositol change and annualized glutamate change (r=0.58, P=0.006) in FES (Supplementary Figure 3).

Myo-inositol and Cannabis

Cannabis use may affect many brain metabolites. Statistical correction for this variable is fraught with challenges. As cannabis use was absent across all healthy controls, there was no sufficient variance to adjust for without eliminating the group effect. The difference in myo-inositol change between self-admitted cannabis users and non-users in the patient group is shown in Supplementary Figure 4. We did not see a statistically significant difference, which may have resulted from a type-II error.

Patient Antipsychotic Drug Information

Supplementary Table 3 shows a detailed list of antipsychotic medication information for each patient.

Supplementary Table 1

Baseline and follow-up scan dates for all volunteers.

HC healthy controls, *FES* first-episode schizophrenia

Subject	Group	Baseline Scan Date	Follow-up Scan Date
001	FES	2017-03-28	2017-09-08
002	FES	2017-04-13	2017-10-06
003	FES	2017-04-21	2017-09-19
004	FES	2017-05-17	2017-10-25
005	FES	2017-06-16	2017-12-12
006	FES	2017-06-16	2018-02-06
007	FES	2017-06-19	2017-11-14
008	FES	2017-06-21	2018-02-07
009	FES	2017-06-23	2017-12-15
010	FES	2017-06-27	2017-12-12
011	FES	2017-08-03	2018-01-10
012	FES	2017-08-09	2018-01-16
013	FES	2017-08-18	2018-01-25
014	FES	2017-10-18	2018-04-25
015	FES	2017-10-20	2018-05-03
016	FES	2017-11-07	2018-07-06
017	FES	2018-01-19	2018-04-27
018	FES	2018-01-23	2018-06-04
019	FES	2018-02-16	2018-10-04
020	FES	2018-02-23	2018-09-07
021	FES	2018-03-27	2018-11-23
022	HC	2017-02-10	2017-10-04
023	HC	2017-04-05	2018-02-09
024	HC	2017-04-07	2018-02-21
025	HC	2017-07-26	2018-04-27
026	HC	2017-09-15	2018-03-09
027	HC	2017-10-25	2018-04-25
028	HC	2017-10-27	2018-04-30
029	HC	2017-11-02	2018-05-04
030	HC	2017-11-24	2018-06-01
031	HC	2017-12-06	2018-09-10

Supplementary Figure 1

(A) Axial, (B), coronal, and (C) sagittal views of MRS voxel (red square) in the dorsolateral anterior cingulate cortex (ACC) for myo-inositol measurements.

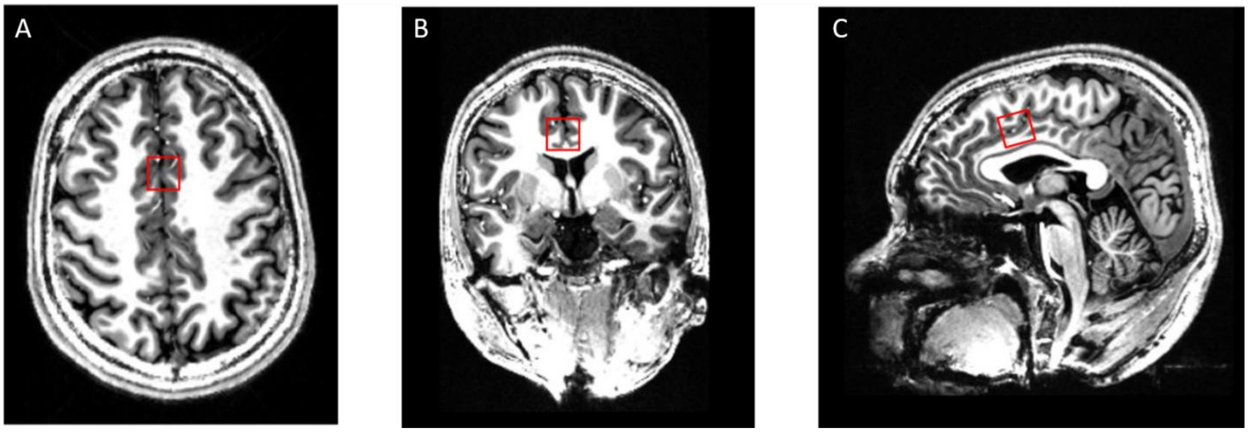

Supplementary Table 2

Mean Metabolite Concentration and CRLB

	$[HC]_{baseline}$	$[HC]_{FUP}$	$CRLB_{HC\_baseline}$	$CRLB_{HC\_FUP}$	$[FES]_{baseline}$	$[FES]_{FUP}$	$CRLB_{FES\_baseline}$	$CRLB_{FES\_FUP}$
**NAA**	11.37 (2.03)	10.82 (1.05)	1.03 (0.34)	0.93 (0.20)	10.44 (1.03)	10.71 (1.77)	1.26 (1.08)	1.01 (0.29)
**Creatine**	9.31 (1.32)	8.77 (0.80)	1.28 (0.33)	1.34 (0.29)	8.85 (0.82)	9.08 (1.50)	1.28 (0.39)	1.38 (0.34)
**Choline**	2.64 (0.49)	2.54 (0.28)	1.90 (0.66)	1.97 (0.47)	2.47 (0.29)	2.61 (0.50)	1.81 (0.48)	1.95 (0.58)
**Myo-inositol**	5.45 (0.99)	5.02 (0.61)	3.94 (1.39)	4.26 (1.07)	4.62 (0.64)	5.01 (1.11)	4.21 (1.12)	4.37 (1.04)
**Scyllo-inositol**	0.34 (0.13)	0.33 (0.12)	18.42 (3.21)	19.83 (5.36)	0.35 (0.11)	0.37 (0.23)	17.07 (4.84)	21.54 (10.94)
**Glutamate**	7.25 (1.34)	6.86 (0.73)	3.43 (1.27)	3.55 (0.89)	6.51 (0.64)	6.49 (1.29)	3.52 (1.20)	3.96 (1.12)
**Glutamine**	1.10 (0.36)	0.98 (0.31)	20.89 (7.01)	24.93 (11.56)	1.06 (0.32)	1.09 (0.40)	19.67 (7.18)	22.91 (9.56)
**Glutathione**	1.71 (0.36)	1.75 (0.23)	10.92 (5.67)	9.55 (1.18)	1.64 (0.25)	1.63 (0.32)	9.99 (3.37)	10.75 (2.60)

CRLB Cramer-Rao Lower Bound, *HC* healthy controls, *FES* first-episode schizophrenia, *FUP* follow-up, *NAA* N-acetyl aspartate, *SD* standard deviation

Note: Mean (SD) concentration and CRLB (SD) units are measured in mM and %, respectively. Only those metabolites with CRLB ≤ 50% were included in this table (all CRLB outliers of ≥ 50% were removed).

Supplementary Figure 2

Sample fitted spectrum of a single participant. Fit spectrum (bolded) is overlaid on the raw spectrum with the residual spectrum displayed above. Individual component spectra of the 8 metabolites reported in Supplementary Table 1 are displayed below.

Abbreviation: GSH, glutathione; Gln, glutamine; Glu, glutamate; Scyllo, scyllo-inositol; Myo, myo-inositol; Cho, choline; Cr, creatine; NAA, N-acetyl aspartate.

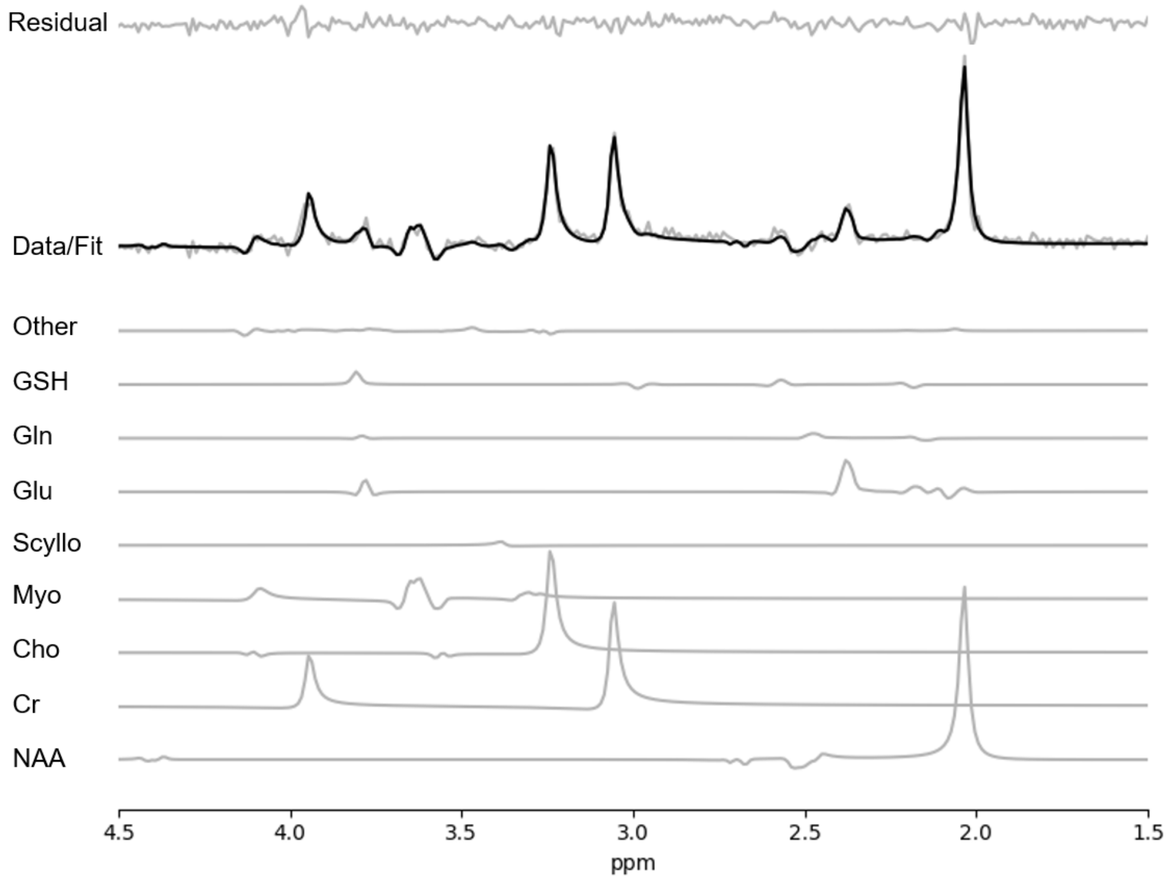

Supplementary Figure 3

Plot of annualized myo-inositol change against annualized glutamate change in FES. Each point represents an individual patient's annualized myo-inositol and glutamate change. The line of best fit is also represented by the solid black line overlayed on the plot.

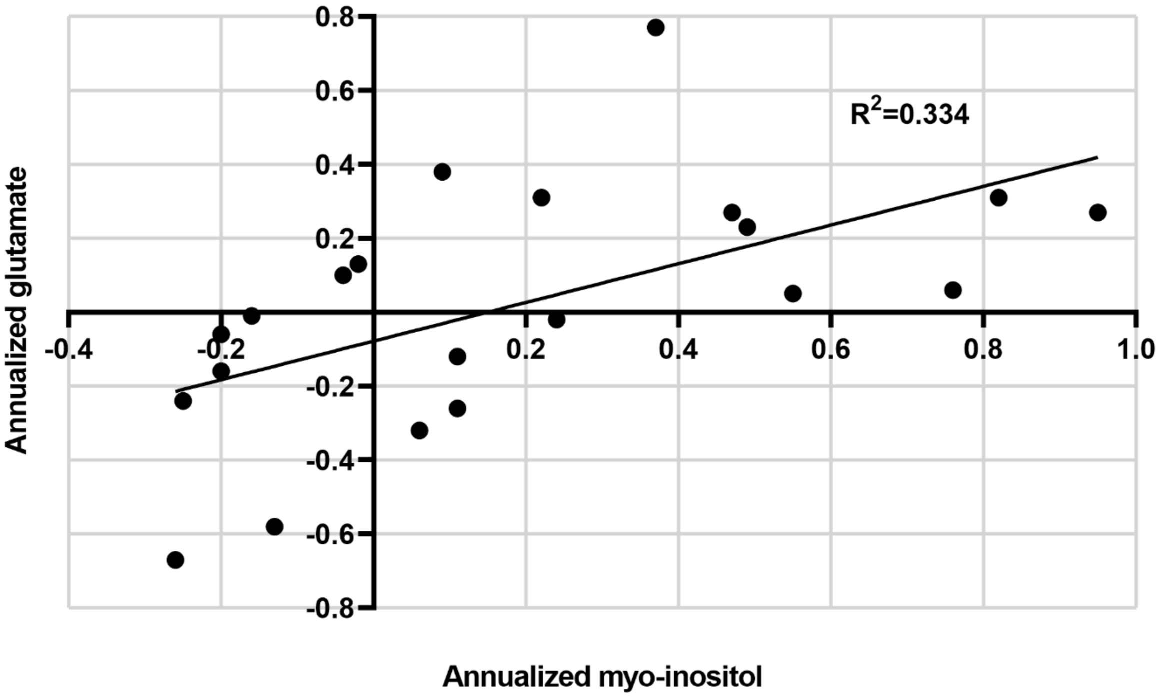

Supplementary Figure 4

Estimation plot of the difference in myo-inositol change between self-admitted cannabis users and non-users.

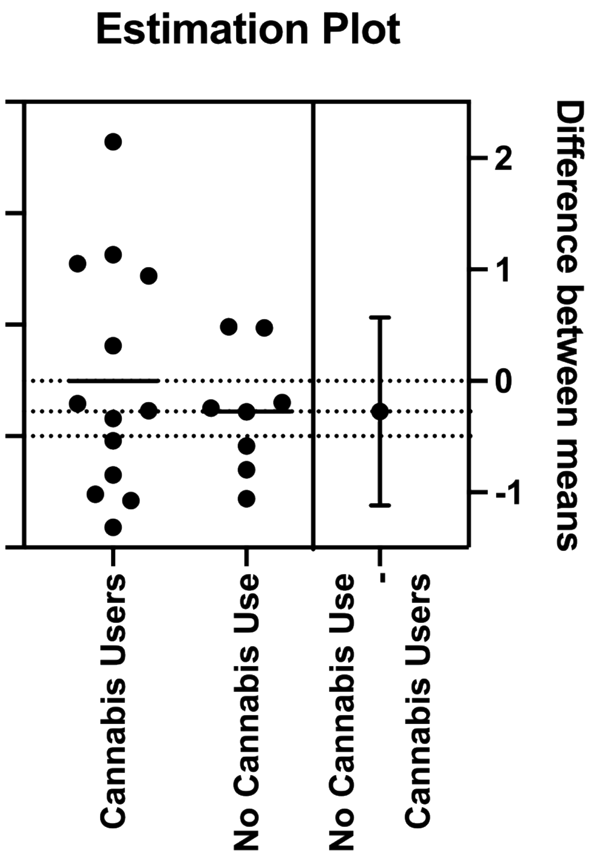

Supplementary Table 3

Detailed list of antipsychotic medication, dose, days administered, and defined daily dose (DDD) at the 6-month follow-up for each patient.

Subject	Antipsychotic	Dose (mg)	Days	DDD (days)
**001**	Abilify	5	10	3.333
	Abilify	10	56	37.33
	Abilify Maintena	300 q28	112 (4 full)	92.27
			**TOTAL:**	**132.2**
**002**	Abilify	2	11	1.466
	Abilify	10	41	27.33
	Invega	6	7	7
	Invega	9	75	112.5
	Invega	12	16	32
	Invega Sustenna	75mg	1 dose	30
	Invega Sustenna	150	11	

			**TOTAL:**	**232**
**003**	Olanzapine	10	5	5
	Olanzapine	7.5	3	2.25
	Invega	6	34	34
	Invega Sustenna	75	1 dose	30
	Abilify	5	7	2.33
	Abilify	10	62	41.33
			**TOTAL:**	**114.91**
**004**	Invega	6	71	71
			**TOTAL:**	**71**
**005**	Olanzapine	10	1	1
	Olanzapine	20	13	26
	Olanzapine	25	56	140
	Olanzapine	20	40	80
	Rexulti	1	6	2
	Olanzapine	10	13	13
	Rexulti	2	6	4
	Olanzapine	5	7	3.5
			**TOTAL:**	**269.5**
**006**	Abilify	5	20	6.66
	Abilify Maintena	300 q28	198	160
			**TOTAL:**	**167**
**007**	Invega	6	121	121
			**TOTAL:**	**121**
**008**	No Medication			0
			**TOTAL:**	**0**
**009**	Olanzapine	5	7	3.5
	Olanzapine	10	3	3
	Risperidone	1	4	0.8
	Risperidone Consta	37.5 q14	84	84
	Risperidone Consta	50 q14	8	10.56
			**TOTAL:**	**101.86**
**010**	Olanzapine	10	5	5
			**TOTAL:**	**5**

**011**	Invega Sustenna	150	6	60
	Invega Sustenna	75	160	160
			**TOTAL:**	**220**
**012**	Risperidone	1.5	17	12.75
	Risperidone	2	23	23
	Risperidone	3	38	57
	Invega Sustenna	100 q28	63	90
			**TOTAL:**	**182.75**
**013**	Abilify	5	86	28.67
	Olanzapine	10	1	1
	Invega Sustenna	150 q28	51	109.29
			**TOTAL:**	**139**
**014**	Rexulti	0.5	7	1.16
	Rexulti	1	21	7
	Rexulti	3	61	61
	Rexulti	1	113	37.6
			**TOTAL:**	**106.826**
**015**	Abilify	5	88	29.333
	Abilify	10	21	14
	Abilify	15	5	5
			**TOTAL:**	**48.333**
**016**	Olanzapine	10	255	255
			**TOTAL:**	**255**
**017**	No medication		0	0
			**TOTAL:**	**0**
**018**	Risperidone	1	6	1.2
	Risperidone	2	62	25
	Invega Sustenna	150 q28	138	273.81
			**TOTAL:**	**300**
**019**	Olanzapine	5	4	2
	Olanzapine	7.5	90	67.5
	Olanzapine	2.5	83	20.75
			**TOTAL:**	**90.25**
**020**	Risperidone	2	7	2.8
	Abilify	5	5	1.67
	Abilify	7	25	11.67

	Risperidone	1	10	2
	Maintena	400q28	28	29.19
	Invega	9	22	33
	Invega Sustenna	150 q28	76	150.79
	Invega Sustenna	100 q28	84	111.1
			**TOTAL:**	342.22
**021**	Abilify	5	19	6.33
	Abilify	7	20	9.33
	Abilify	15	44	44
	Maintena	300q28	56	43.79
	Maintena	200q28	112	58.39
			**TOTAL:**	**161.95**

References

1. Rothermundt, M. *et al.* Glial cell activation in a subgroup of patients with schizophrenia indicated by increased S100B serum concentrations and elevated myo-inositol. *Prog. Neuro-Psychopharmacology Biol. Psychiatry* **31**, 361–364 (2007).
2. Bitsch, A. *et al.* Inflammatory CNS demyelination: Histopathologic correlation with in vivo quantitative proton MR spectroscopy. *Am. J. Neuroradiol.* **20**, (1999).
3. Castillo, M., Smith, J. K. & Kwock, L. Correlation of myo-inositol levels and grading of cerebral astrocytomas. *Am. J. Neuroradiol.* **21**, (2000).
4. Hattingen, E. *et al.* Myo-inositol: A marker of reactive astrogliosis in glial tumors? *NMR Biomed.* **21**, 233–241 (2008).
5. Chen, S. Q. *et al.* Role of myo-inositol by magnetic resonance spectroscopy in early diagnosis of Alzheimer’s disease in APP/PS1 transgenic mice. *Dement. Geriatr. Cogn. Disord.* **28**, (2009).
6. Chen, S. Q. *et al.* 1H-MRS evaluation of therapeutic effect of neural stem cell transplantation on Alzheimer’s disease in AβPP/PS1 double transgenic mice. *J. Alzheimer’s Dis.* **28**, (2012).

7. Tkáč, I. & Gruetter, R. Methodology of 1H NMR spectroscopy of the human brain at very high magnetic fields. *Appl. Magn. Reson.* **29**, 139–157 (2005).

8. Near, J. *et al.* Frequency and phase drift correction of magnetic resonance spectroscopy data by spectral registration in the time domain. *Magn. Reson. Med.* **73**, 44–50 (2015).

9. Bartha, R., Drost, D. J., Menon, R. S. & Williamson, P. C. Spectroscopic lineshape correction by QUECC: Combined QUALITY deconvolution and eddy current correction. *Magn. Reson. Med.* **44**, 641–645 (2000).

10. van den Boogaart, A., Ala-Korpela, M., Jokisaari, J. & Griffiths, J. R. Time and frequency domain analysis of NMR data compared: An application to 1D 1H spectra of lipoproteins. *Magn. Reson. Med.* **31**, 347–358 (1994).

11. Bartha, R., Drost, D. J. & Williamson, P. C. Factors affecting the quantification of short echo in-vivo1H MR spectra: Prior knowledge, peak elimination, and filtering. *NMR Biomed.* **12**, 205–216 (1999).

12. Wong, D. MRI Investigations of Metabolic and Structural Brain Changes in Alzheimer's Disease and Vitamin D Deprivation. (2019).
